# USING ACCESSIBLE TECHNOLOGY TO SUPPORT CAREGIVERS OF PERSONS WITH DEMENTIA

**DOI:** 10.1093/geroni/igz038.910

**Published:** 2019-11-08

**Authors:** Katie Maslow, Scott A Trudeau, Sara J Czaja

**Affiliations:** 1 Gerontological Society of America, Washington, District of Columbia, United States; 2 American Occupational Therapy Association, Bethesda, Maryland, United States; 3 Weill Cornell Medicine, New York, New York, United States

## Abstract

There is widespread enthusiasm about the potential of technology in general to support persons living with dementia and their families and other caregivers. At the same time, recommendations from the 2017 National Research Summit on Care, Services, and Supports for Persons with Dementia and their Caregivers emphasize the need for research to develop, evaluate, and disseminate specific technologies that can achieve meaningful benefits for well-defined subgroups of persons living with dementia and their caregivers, including individuals from diverse populations and individuals who live and receive care in various settings. This symposium focuses on specific home-based technologies to help family caregivers of community-living persons with dementia. Our three speakers will talk about research results for three different technology-related interventions, including: use of home video telehealth visits to help family caregivers provide effective dementia care and provide medical management; use of home video assessments by occupational therapists to help family caregivers increase home safety for community-living persons with dementia; and approaches for making a self-paced Home Safety Toolkit available to family caregivers of community-living veterans with dementia. Each speaker will report both positive outcomes, including family caregiver satisfaction, and barriers encountered in delivering the interventions. Such barriers include difficulties with the technologies as well as caregiver reluctance to change and costs that were not covered by health care insurance or health systems. Our discussant will respond to the presentations and solicit audience questions and discussion.

